# Impact of diabetes on male sexual function in streptozotocin-induced diabetic rats: Protective role of soluble epoxide hydrolase inhibitor

**DOI:** 10.1016/j.biopha.2019.108897

**Published:** 2019-05-15

**Authors:** Nathani Minaz, Rema Razdan, Bruce D. Hammock, Somdutt Mujwar, Sumanta Kumar Goswami

**Affiliations:** a Department of Pharmacology, Al-Ameen College of Pharmacy, Bangalore, Karnataka, India; b Department of Entomology and Nematology, and Comprehensive Cancer Center, University of California, Davis, CA, USA; c Institute of Pharmaceutical Research, GLA University, 17km Stone, NH-2, Mathura-Delhi Road P.O. Chaumuhan, Mathura, 281 406, Uttar Pradesh, India

**Keywords:** Diabetes-induced sexual dysfunction, sEH inhibitor *t*-TUCB, Testosterone, Sperm count, Corpus cavernosum

## Abstract

Diabetes-induced male sexual dysfunction is associated with endothelial dysfunction. Inhibition of soluble epoxide hydrolase (sEH) is known to improve endothelial function in diabetes. Therefore, we hypothesized that sEH inhibitor (sEHI), [trans-4-{4-[3-(4-trifluoromethoxyphenyl)-ureido]cyclohexyloxy}benzoic acid] / *t*-TUCB can restore the male sexual function in diabetic rat. After one week of administration of diabetogenic agent STZ (52 mg/kg i.p) injection, diabetic rats were treated with *t*-TUCB (0.1 and 0.3 mg/kg, p.o) or vehicle for 8 weeks. The sexual behaviour parameters of the animals were evaluated at the end of dosing period. The levels of testosterone and glucose in serum, and sperm were quantified. Effect of treatment on weight of reproductive organs and histopathology of penile tissue was evaluated. Diabetes had a negative effect on male sexual function, weight of sexual organs and production of sperm with a parallel decrease in the level of testosterone. The sEHI, *t*-TUCB, significantly preserved the sexual function and minimized an increase in the level of blood glucose in diabetic rats. It also prevented a decrease in the level of testosterone and sperm in diabetic rats, in comparison to diabetic control rats. Further, diabetes induced distortion of corpus cavernosum was attenuated by *t*-TUCB. Based on our findings, sEHI may delay the development of sexual dysfunction in diabetes.

## 1. Introduction

Sexual dysfunction (SD) refers to a problem or a group of problems occurring during any phase of the sexual response cycle, including desire, physical pleasure, arousal or orgasm, which prevents the individual from experiencing satisfactory sexual activity [1]. It can contribute to psychological problems such as feelings of inadequacy, frustration, loss of self-esteem, and despair. Erectile dysfunction (ED) is one of the sexual dysfunctions which, primarily, results from impairment of penile vascular and smooth muscle relaxation in multiple pathological conditions including diabetes. A threefold increase in the risk of ED was documented in diabetic men compared with non-diabetic men [2,3]. One of the major pathophysiological mechanisms underlying diabetic ED is endothelial dysfunction [4].

Endothelial dysfunction is manifested in diabetes due to alterations in many signal transduction pathways responsible for the stimulation of penile vascular and smooth muscle relaxation. An enhanced destruction of nitric oxide (NO) due to an increase in oxidative stress, and a decrease in the release of endothelium-derived hyperpolarizing factors (EDHFs) are few of the mechanisms for development of diabetic ED [5]. In addition to NO, EDHFs contribute significantly to the dilation of penile arteries [6]. Epoxyeicosatrienoic acids (EETs) derived from the arachidonic acid act as EDHFs [7,8]. EETs are rapidly hydrolyzed to less bioactive diols by the soluble epoxide hydrolase (sEH). Data from animal studies have shown that inhibitors of sEH attenuate endothelial dysfunction in diabetes [9]. Yousif et al reported that CDU, a sEH inhibitor, regulates the tone of corpus cavernosum smooth muscle [10]. Earlier, we reported that *Moringa oleifera* (seed) extract inhibits sEH and augments sexual function in healthy rats [11]. Based on these studies, we hypothesized that inhibition of sEH might have a positive impact on sexual function in diabetes.

## 2. Materials and methods

### 2.1. Chemicals and assay kit

The soluble epoxide hydrolase inhibitor, *t*-TUCB, was synthesized as described previously [12]. Streptozotocin/STZ (MP Biomedical Pvt. Ltd.), ketamine hydrochloride (Neon Laboratories Limited, India), xylazine (Indian Immunologicals Limited, India), diethyl stilbestrol (Penta Pharmaceuticals, India), progesterone (Sun Pharmaceutical Ind. Ltd, India) and assay kit for estimation of blood glucose (Autospan diagnostic, India) level were procured.

### 2.2. Animal

Twentyfour, in-bred sexually active male Wistar rats weighing (250 ± 5.00) g and an equal number of female rats were obtained from the central animal house, Al-Ameen College of Pharmacy, Bangalore. The rats were harboured in polypropylene cages and housed in a well-ventilated animal house under following standard laboratory conditions: temperature, 22 ± 3 °C; 12h light/12h dark cycle; humidity, 45–50%. Ethical clearance was obtained from the institutional animal ethics committee and the procedures were performed according to the recommendations and guidelines of Committee for the Purpose of Control and Supervision of Experiment on Animals (CPCSEA), India.

### 2.3. Induction of diabetes

Sexually active male rats were divided into four groups. Six rats were considered as normal control and treated with vehicle for 8 weeks. Another 18 rats were induced diabetes. Diabetes was induced via a single i.p. dose of 52 mg/kg STZ that was freshly prepared in 0.1 mM citrate buffer, pH 4.5 and used within 10 min of preparation [13]. All rats receiving STZ were given a 10% sucrose solution in the first 48 h after injection to prevent hypoglycaemia. After 72 h of STZ administration, rats with fasting serum glucose more than 230 mg/dL were considered as diabetic. Long-acting insulin (3 IU/kg, s.c) was administered twice in a week to diabetic rats to prevent mortality throughout the study. Six diabetic rats were treated with vehicle for 8 weeks and considered as diabetic control. Remaining 12 diabetic rats were equally divided and treated with *t*-TUCB either with 0.1 or 0.3 mg/kg, p.o for eight weeks.

### 2.4. Assessment of mating behaviour

In our study, female rats were used to stimulate male rats for initiating a sexual function. Ovariectomized female rats were used to prevent pregnancy and minimize the number of female rats used in the study [14]. Female rats were paired with multiple male rats on different days during training and actual study. The ovariectomized female rats were artificially brought into oestrus phase by the administration of diethylstilbestrol (1 mg/kg, p.o, administered 2 days before the study) and progesterone (5 mg/kg, s.c., administered 6 h prior to the study) to facilitate mating [15]. It is shown that female rats in the estrous phase allow intromission by exhibiting lordosis posture [16]. Intromission was considered as a marker of erectile function. The male and female rats were trained for sexual behaviour study before induction of diabetes and treatment [17]. The male and female rats who did not involve in sexual activity even after 10 min of pairing during training were removed from the study. The male rats were allowed to acclimatize to the environment for 10 min in a wooden cage (45 × 50 × 35 cm) having glass covering and illuminated by red light and then a female rat was placed in the cage and following sexual behaviour was observed for 30 min [18]: Mount latency(ML), time from the introduction of female rat into the cage of the male rat up to the first mount; Intromission latency (IL), time from the introduction of the female rat up to the first intromission by the male rat; Mount frequency (MF), number of mounts before ejaculation; Intromission frequency (IF), number of intromission before ejaculation; Ejaculation latency (EL), time from the first intromission of a series up to the ejaculation); Post-ejaculatory interval (PEI), time from the first ejaculation up to the next intromission by the male rat.

### 2.5. Estimation of biochemical parameters

Blood was collected from the heart of an anesthetized rat and serum was separated. Levels of testosterone and glucose in serum were quantified using commercial diagnostic kits according to test procedures outlined in the manual of the kits [19].

### 2.6. Assessment of sperm count and morphology

At the end of the study, male rats were sacrificed by an overdose of anaesthesia. Epididymis, testis and penile tissue from each rat were excised and weighted. Epididymis was used for sperm count and morphology studies, whereas penile tissue was stored in 10% formalin solution for histopathology. Sperm count and analysis were performed by the methods reported earlier [20]. Abnormal sperms (coiled sperms, tailless sperms, sperms with a bent neck, midpiece and tails) were observed using an eosin staining method. Two hundred sperms per animal were examined microscopically at 40x magnification and numbers of abnormal sperms were counted to calculate the percentage of abnormal sperms.

### 2.7. Histopathology of penile tissue

Excised shafts of penile tissues were fixed in a 10% formalin-salines olution for 24 h followed by washing in distilled water for half an hour. Then tissues were stored in a centrifuge tube containing 70% ethanol till processing. Paraffin sections of tissue (5 μm thickness) were obtained and H&E staining was performed. The pathologist was blinded to the grouping [21].

### 2.8. Statistical analysis

Values are expressed as mean ± S.E.M. Statistical significance with respect to diabetic control was evaluated using one-way ANOVA followed by Dunnet’s test using Graph Pad Prism 5 (Version 5.0, GraphPad Software Inc., San Diego, CA).

## 3. Results

### 3.1. Inhibition of sEH preserves sexual function in diabetic rats

**Fig. 1.**
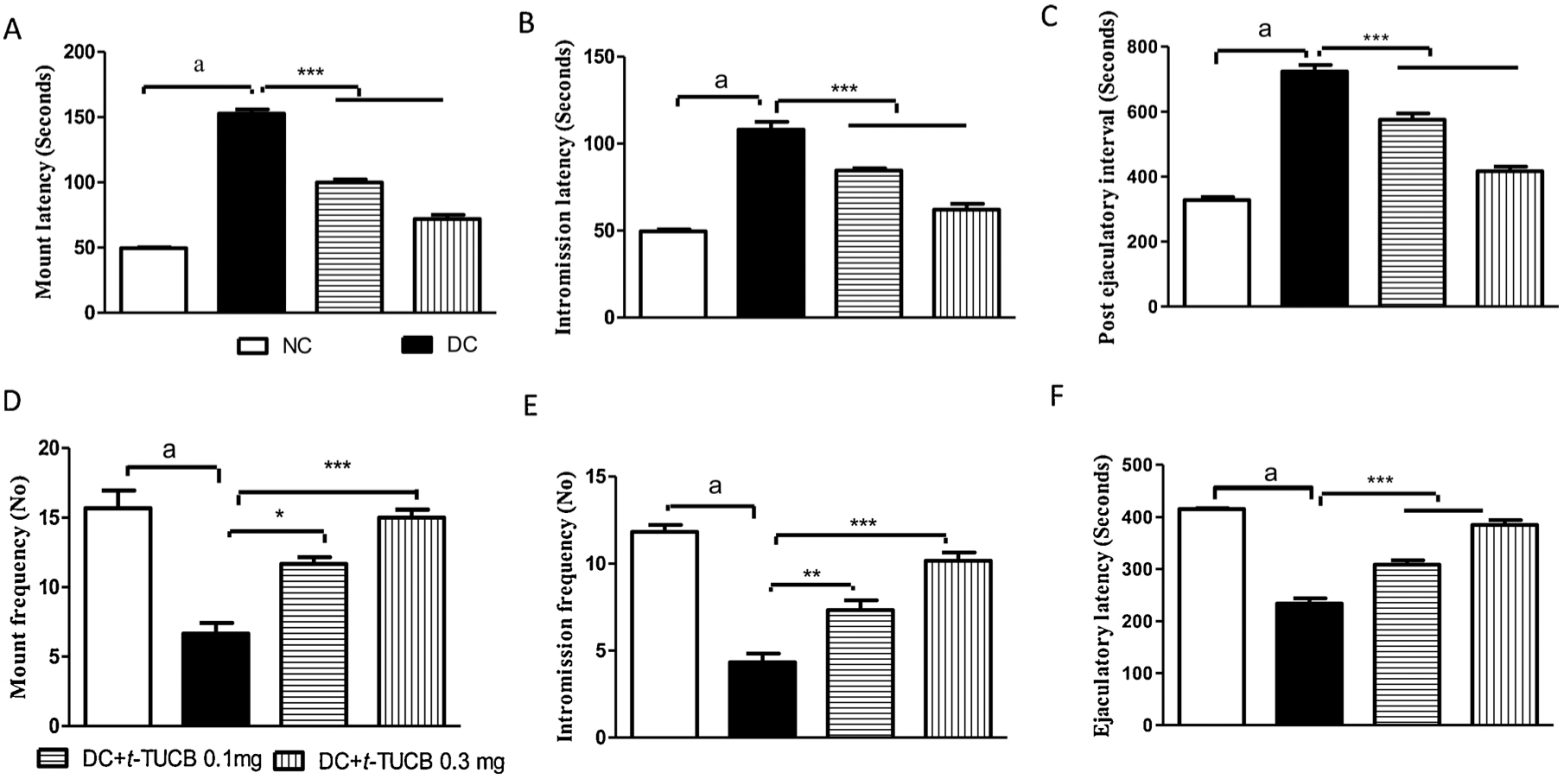
The *t*-TUCB decreases diabetes-induced sexual dysfunction. An increase in the mount latency (A), intromission latency (B) and postejaculatory interval (C), but a decrease in the mount frequency (D), intromission latency (E) and ejaculatory latency (F) was observed in diabetic rats, in comparison to normal control rats. Diabetes-induced significant (^a^ p < 0.001) sexual dysfunction is evident due to increase in mount latency, intromission latency, post ejaculatory interval and decrease in mount frequency, intromission frequency and ejaculatory latency, in comparison to normal control animals. Treatment of *t*-TUCB significantly preserved sexual function, in comparison to diabetic control rats. * p < 0.05, ** p < 0.05, *** p < 0.001 when compared with diabetic control rats. NC: normal control, DC: diabetic control. Values are expressed as mean ± S.E.M. of six observations.

The chronic hyperglycaemia in sexually active male rats significantly reduced MF, IF, and EL, and increased ML, IL and PEI compared to normal rats. Treatment with *t*-TUCB restored the sexual function in diabetic rats as evident by an increasing in MF, IF, and EL and a decrease in ML, IL and PEI compared to diabetic control rats in a dose dependent manner (Fig. 1).

### 3.2. Inhibition of sEH reduces serum glucose level in diabetic rats

**Fig. 2.**
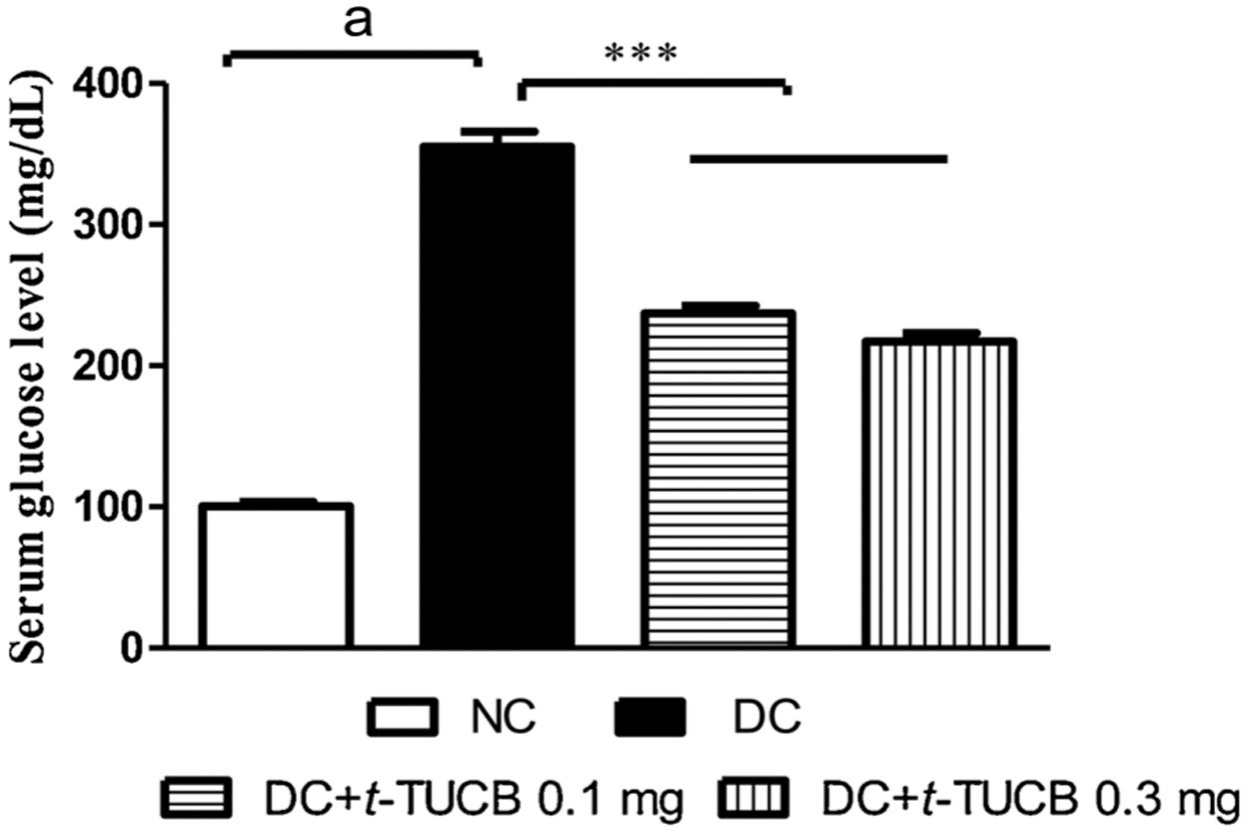
The *t*-TUCB minimizes an increase in the level of glucose in the serum of diabetic rats. Serum glucose level was significantly increased in the diabetic rats (^a^ p < 0.001) in comparison to the normal control rats. Treatment of *t*-TUCB prevented an increase in the serum glucose level (*** p < 0.001), in comparison to diabetic rats. NC: normal control, DC: diabetic control.

A significant increase in the level of glucose in the serum was observed in diabetic rats compared to normal rats. Treatment with *t*-TUCB decreased serum glucose level compared to diabetic rats in a dose dependent manner (Fig. 2).

### 3.3. Inhibition of sEH prevents a decrease in the weight of sex organs from diabetic rats

A decrease in the weight of testes, and penis were observed in the diabetic rats compared to normal healthy rats. Treatment with *t*-TUCB in diabetic male rats prevented a significant decrease in the weight of testes, and penis compared to diabetic rats (Table 1).

### 3.4. Inhibition of sEH minimizes a reduction in the total sperm count and abnormality in diabetic rats

**Fig. 3.**
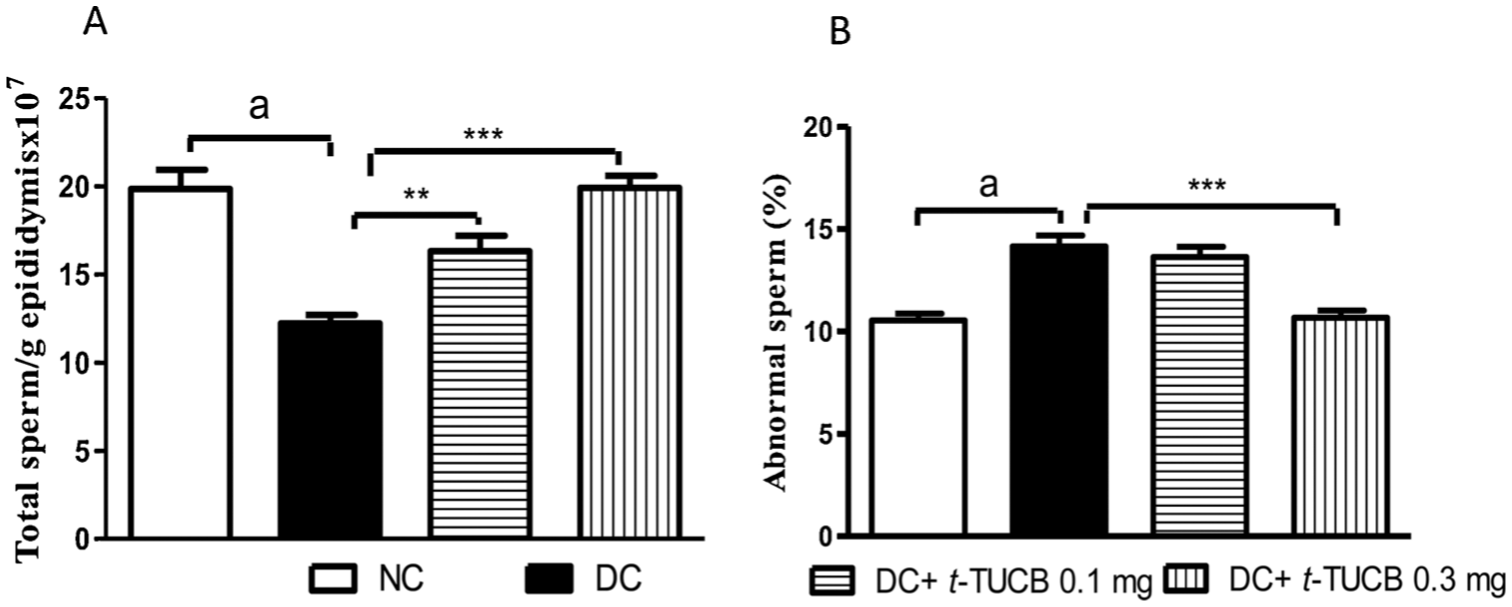
The *t*-TUCB improves sperm count and abnormal sperm of diabetic rats. Sperm count (A) was significantly decreased while % abnormal sperm (B) was significantly increased in the diabetic rats (^a^ p < 0.001), in comparison to the normal control rats. Treatment with *t*-TUCB minimized an increase in abnormal sperm and a decrease in sperm count in diabetic rats, in comparison to diabetic control rats. ** p < 0.05, *** p < 0.001 when compared with diabetic control rats. NC: normal control, DC: diabetic control. Values are expressed as mean ± S.E.M. of six observations.

A significant reduction in sperm count was observed in the diabetic rats with more abnormality in sperm morphology compared to normal rats. Treatment with *t*-TUCB prevented a decrease in the total sperm count and abnormality in sperm morphology compared to diabetic control rats in a dose dependant manner (Fig. 3).

### 3.5. Inhibition of sEH preserves serum testosterone level in diabetic rats

The level of testosterone in serum was decreased in diabetic rats compared to normal rats. Treatment with *t*-TUCB minimizes a decrease in the level of testosterone compared to diabetic control rats in a dose dependant manner (Table 1).

### 3.6. Inhibition of sEH preserves the anatomical architecture of penile tissue in diabetic rats

**Fig. 4.**
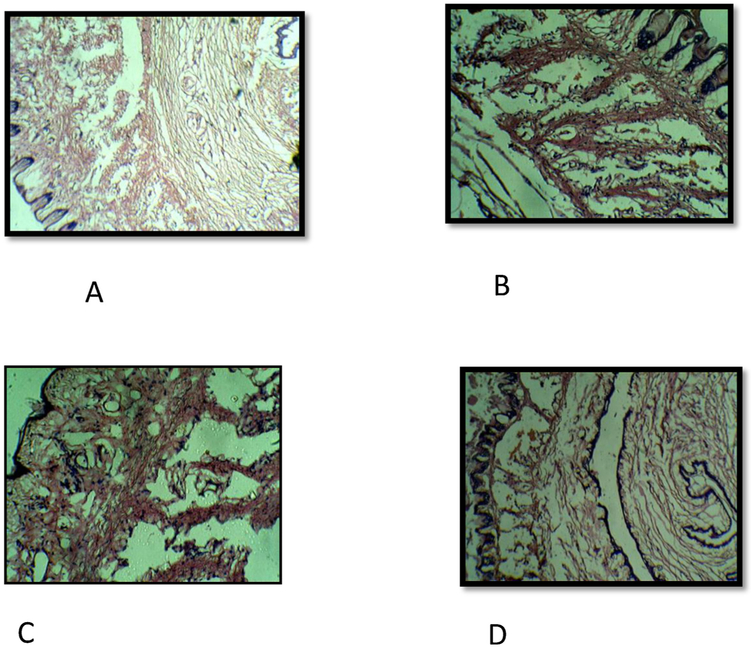
The *t*-TUCB improves pathological changes of penile tissue in diabetic rat. Histopathology of penile tissue of normal rat (A) revealed a dilated corpus cavernosum lined with prominent endothelium, smooth muscle and fibroelastic connective tissue. Penile tissue of diabetic rat (B) revealed collapsed cavernous spaces and depleted endothelium. Penile tissue of diabetic rat treated with *t*-TUCB (0.1 mg/kg) (C) revealed few distortions of cavernous spaces lined by smooth muscle and connective tissue. Penile tissue of diabetic rat treated with *t*-TUCB 0.3 mg/kg (D) revealed cavernous spaces lined by endothelium with intact smooth muscle and fibroelastic connective tissue.

Histopathology of penile tissue of normal rat revealed the dilated corpus cavernosum, lined by endothelium and surrounded by smooth muscle, fibro-elastic connective tissue, enclosed by tunica albuginea. The penile tissue of diabetic rat was distorted anatomically with collapsed cavernous spaces and a prominent reduction in the endothelium. The penile tissue of diabetic rat treated with *t*-TUCB 0.1 mg/kg had fewer distortion of smooth muscle and connective tissue. The penile tissue of diabetic rat treated with *t*-TUCB 0.3 mg/kg had almost normal cavernous spaces lined by endothelium with intact smooth muscle and fibroelastic connective tissue (Fig. 4).

## 4. Discussion

With the increasing prevalence of diabetes, a large number of males suffer from diabetic ED which has a severe impact on health and life [22]. Diabetic endothelial dysfunction is the common denominator leading to vascular ED. Interestingly, cytochrome P450 (P450) generates EETs in the endothelial cells which are known to relax endothelial cells via activation of calcium-activated and voltage-gated potassium ion channel (BK_Ca_) through a cAMP and PKA mediated mechanism. EETs stabilized by sEH inhibitors are known to alleviate endothelial dysfunction [23-26]. Jin et al reported that P450 2B, 2C and 2 J are present in rat corpus cavernosum. They further demonstrated that P450 2C which generates EETs efficiently than other P450 s are localized in cavernosal endothelial cells and reported that 11, 12-EET is one of the main EETs generated in the corpus cavernosa. Administration of EETs antagonist reduced major pelvic ganglion stimulation-induced increase in the intracavernous pressure, a marker of penile erection [27]. Recently, several researchers have reported that chronic therapy with *t*-TUCB significantly decrease the incidence of cardiovascular disease and protects endothelial function in diabetic animals [28,29]. Therefore, we hypothesized that *t*-TUCB treatment may have a beneficial effect against STZ-induced diabetic ED. STZ-induced diabetes in animals is an excellent model to study diabetic ED.

The chronic hyperglycaemia clearly affected sexual function in male rats. Mounting behaviour represents sexual provocation, while intromission indicates the efficiency of erection and penile orientation [30]. Increase in ML and decrease in MF of the diabetic rats in our study indicates a decrease in sexual desire, while an increase in IL and decrease in IF indicates an erectile dysfunction. A significant increase in MF and IF, and a decrease in ML and IL in diabetic rats by *t*-TUCB suggests that it has preserved the sexual performances in diabetic rats. Ejaculatory latency and post-ejaculatory interval reveal duration of erection and time taken for regaining an erection after first ejaculation, respectively [31,32]. A decrease in ejaculatory latency and increase in post-ejaculatory interval in diabetic rat confirms impairment of penile erection. A reduction in PEI by *t*-TUCB suggests that copulatory efficiency of diabetic male rats increased with the treatment. The treatment with *t*-TUCB in diabetic rat restored the sexual excitement and desire during the period of sexual interaction with active female rats. Inhibition of sEH preserves vasodilator EETs which has a therapeutic benefit in improving endothelial function in diabetes and cardiometabolic diseases [28,33]. Extract of the plant *Moringa oleifera*, a natural inhibitor of sEH, is reported to relax penile tissue *ex vivo* and increase intracavernous pressure *in vivo* [11]. Therefore, inhibition of sEH is, at least, responsible for the alleviation of sexual dysfunction in diabetic rats.

A decrease in the level of testosterone in diabetic rat could be considered as one of the contributing factors responsible for an overall decrease of sexual performance, because testosterone regulates nearly every component of erectile function, from sexual desire to penile erection involving co-ordinated activity of pelvic ganglions, smooth muscle and endothelial cells of the corpora cavernosa [19]. Castration is known to decrease the nitric oxide synthase immunoreactivity in medial preoptic nucleus and nucleus of stria terminalis, suggesting a role of testosterone in controlling production of NO [34]. Our results are in support with previously published report which revealed that induction of diabetes by high doses of STZ in male leads to a reduction in testosterone level [35]. A decrease in the function of both Leydig (testosterone producing cell) and sertoli (spermatogenesis) is associated with a reduction in the secretion of insulin [36,37]. The treatment with *t*-TUCB prevented a decrease in the level of testosterone in diabetic rats which could be due to the anti-hyperglycemic effect of sEH inhibitor as reported by us earlier [38]. This study demonstrated an importance of sEHI in regulating the level of testosterone in diabetes. Earlier, we had reported that the level of testosterone was decreased in the male mice lacking sEH [39]. Similarly, the activity of sEH in the kidney and liver of the castrated male mice was decreased. As expected, administration of testosterone to castrated mice increased the activity of sEH in kidney and liver in comparison to sham mice. Interestingly, testosterone also affects the spermatogenesis [40].

Further, diabetic rat exhibited low sperm count and more abnormality in sperm morphology. The mechanism for impaired spermatogenesis is through hyperglycaemia-induced oxidative stress in testis [41]. The increased oxidative stress in diabetes mellitus favours peroxidation of polyunsaturated fatty acids (PUFA) in the sperm cell membrane. This increased lipid peroxidation causes DNA damage in sperm cell [42]. Treatment with antioxidant is reported to increase live sperm percentage and decrease sperm morphological damage [43]. The sEH inhibitor, *t*-TUCB was reported to possess anti-oxidant property [29]. Therefore, *t*-TUCB might have decreased sperm damage my reducing oxidative stress in diabetes. Even sexual dysfunction in diabetes is associated with oxidative stress [44,45]. and inflammation [46]. A reduction in oxidative stress [47] and inflammation [48] is known to alleviate sexual dysfunction. We have earlier demonstrated the anti-inflammatory and anti-oxidant effect associated with the inhibition of the sEH [49,50,38]. Therefore, *t*-TUCB might have alleviated the sexual dysfunction via reducing oxidative stress and inflammation. Further, the damage to sperm may also be due to the decrease in the level of androgen or hyperglycaemia induced degeneration of seminiferous epithelium and epididymal function [51,52]. The abnormal sperm may be the cause for the decreased fertility in diabetic rats [53]. Administration of *t*-TUCB significantly increased the epididymal sperm count and decreased abnormal sperm percentage, which clearly indicates the spermatogenic efficacy of the sEH inhibitor. Further diabetes also alters the normal architect of penile tissue.

In diabetic rats, corpora cavernosa were collapsed and were lined by depleted endothelium with either the absence or significantly reduced smooth muscle compared to normal rats. Treatment with *t*-TUCB at 0.3 mg/kg in diabetic rats restored the normal anatomical architecture of penile tissue which supports the protective effect of sEH inhibitor in diabetic ED.

Though we demonstrated that *t*-TUCB ameliorates diabetic sexual dysfunction, many questions remain unanswered. Though the sEH inhibitors are known to have pharmacological activity due to an increase the level of EETs [54], the sEH inhibitors also have direct effect [55]. The direct effect of sEHI on corpus cavernosum could not be determined. An *Ex vivo* study demonstrating the effect of sEHI on isolated corpus cavernosum from normal and diabetic rat could have provided conclusive evidence. EET-mimetics have been helpful in delineating the mechanism via which EET or sEHI exhibit protective effect in various disease conditions [56,57]. Use of EET mimetic would have provided conclusive data about the erectogenic potential of *t*-TUCB independent of its effect on the level of blood sugar and testosterone. Evaluating the effect of oxidative stress in testes and corpus cavernosa of study animals could have provided additional information about the role of oxidative stress in diabetic sexual dysfunction and effect of *t*-TUCB in this pathological condition.

## 5. Conclusion

The present study demonstrates that the sEH inhibitor *t*-TUCB ameliorates diabetic sexual dysfunction by minimizing a decrease in the level of testosterone and preserving penile tissue architecture. In addition, the ability of *t*-TUCB to minimize a decrease in sperm count and reduce sperm defects in diabetic rats may also help in increasing the overall reproductive function of diabetics. This inhibitor may be used to alleviate male sexual dysfunction arising due to diabetes.

## Figures and Tables

**Table 1:** Effect of *t*-TUCB on diabetes-induced alteration in level of testosterone, weight of penile and testes.

Parameters	Groups			
	Normal Control	Diabetic Control (DC)	DC + *t*-TUCB 0.1 mg/kg	DC + *t*-TUCB 0.3 mg/kg
Serum testosterone (ng/dL)	96 ± 3^a^	44 ± 4	56 ± 3	71 ± 3***
Penile weight (g)	0.24 ± 0.01^a^	0.18 ± 0.01	0.19 ± 0.01*	0.24 ± 0.01***
Testis weight (g)	0.59 ± 0.02^a^	0.13 ± 0.01	0.33 ± 0.02***	0.51 ± 0.03***

Level of testosterone and weights of penises and testes were reduced in the diabetic rats, in comparison to the normal healthy rats.

a p < 0.001. The sEH inhibitor minimized a reduction in the levels of testosterone in diabetic rats. The *t*-TUCB also minimized a reduction in the weights of penises and testes of diabetic rats, in comparison to diabetic control rats.

* p < 0.05.

*** p < 0.001. Values are presented as mean ± SEM of 4 readings. One-way ANOVA followed by Dunnet’s test was used for statistical calculation. Diabetic control vs diabetic rats treated with sEH inhibitor.
